# Effect of ACTH4-10Pro8-Gly9-Pro10 on anti-inflammatory cytokine (IL-4, IL-10, IL-13) expression in acute spinal cord injury models (male Sprague Dawley rats)

**DOI:** 10.12688/f1000research.127413.2

**Published:** 2025-09-22

**Authors:** Asadullah Asadullah, Abdul Hafid Bajamal, Muhammad Arifin Parenrengi, Agus Turchan, Budi Utomo, I Ketut Sudiana, Eko Agus Subagio

**Affiliations:** 1 Neurosurgery Department, Dr. Soetomo Hospital, Surabaya, East Java, 60286, Indonesia; 2 Neurosurgery Residency Program, Airlangga University, Surabaya, East Java, 60132, Indonesia; 3 Public Health and Preventive Medicine Department, Airlangga University, Surabaya, East Java, 60132, Indonesia; 4 Anatomical Pathology Department, Airlangga University, Surabaya, East Java, 60132, Indonesia

**Keywords:** Spinal Cord Injury, Anti inflammatory cytokine, Neuroprotector, Experimental, Laminectomy

## Abstract

**Background:**

Spinal cord injury (SCI) is a damage to the spinal cord caused mainly by trauma resulting in major motor, sensory and autonomic dysfunctions. Its final neurological outcome is determined by both primary and secondary injury processes. A key component of secondary injury mechanisms after initial trauma is neuroinflammation. A neuroprotective compound, ACTH _4-10_Pro ^8^-Gly ^9^-Pro ^10^ (ACTH _4-10_) also known as semax, has shown neuroprotective and anti-inflammatory properties. ACTH _4-10_ has also been actively used in the treatment of brain ischemia without serious complication reported. Here, we analyzed the effects of ACTH _4-10_ at regulating the inflammatory cascade in SCI by looking at anti-inflammatory cytokine (IL-4, IL-10 and IL-13) levels after acute SCI.

**Method:**

We carried out laminectomies in male Sprague Dawley rats at the second thoracic vertebrae. After laminectomy, we exposed the myelum and created mild SCI models with 20-g, and severe SCI with 35-g aneurysm clips. ACTH _4-10_ was administered intranasally to the treatment group and 0.9% NaCl to the control group (placebo). Both groups were kept alive and terminated at 3 and 6 hours. The tissue sample preparations were fixed in formalin and examined for immunohistochemistry. Quantitative measurement of the cytokines was done in the posterior horn area with specific associated anti-monoclonal antibodies.

**Results:**

Rats with mild SCI that were given ACTH _4-10_ showed greater anti-inflammatory levels at 3 hours post-compression but only IL-10 and IL-13 were elevated significantly at 6 hours. Rats with severe compression in ACTH _4-10_ group showed greater levels of IL-10, IL-13 at 3 hours and IL-4, IL-10 at 6 hours compared with the placebo group.

**Conclusions:**

Administration of ACTH _4-10_Pro ^8^-Gly ^9^-Pro ^10^ intranasal can increase anti-inflammatory cytokine expression in Sprague Dawley rat models with mild and severe SCI. Expression of anti-inflammatory cytokines was greater in mild compression and 3-hour termination. Further research is needed to determine the optimal dose and clinical outcome *in vivo.*

## Introduction

Spinal cord injury (SCI) is a pathological condition that is known to cause severe neurological deterioration resulting in physical dependency, morbidity, mortality and financial burden for decades. ^1^ Even tough significant research has been conducted to develop effective treatments for SCI, until now none of these treatments has shown any meaningful effect to improve functional outcome after injury. ^2^

Demographic data suggest that SCI mainly affects the male population with over 80% cases occurring in males between 25 to 45 year of age. ^3^ Interestingly, not only do males have a higher incidence rate of SCI, but some studies have also shown males tend to have worse recovery rates compared with females. ^4^ This phenomenon is thought to be caused by female sex hormone such as progesterone and estrogen, that have shown modest neuroprotective behaviour in brain injury. ^5^

In general, pathophysiology of traumatic SCI comprises primary and secondary injury. The primary injury is caused by the initial traumatic event such as compression, distraction, laceration and transection; this kind of injury causes irreversible damage to the spinal cord. In contrast, secondary injury, including processes such as neuroinflammation, spinal cord ischemia, and cellular excitotoxicity which begin soon after the initial trauma, give a window of opportunity to prevent further damage of the spinal cord. Therefore, managing secondary injuries remains the main target for SCI treatment. ^6^^,^^7^

Above all the potential threats from secondary SCI, neuroinflammation is thought to be the key component causing many local and systemic consequences. It is known that injury to the spinal cord initiates an inflammatory-mediated response that could alter the repair processes within the nervous system and cause more damage to the nerves. Therefore, managing inflammation is thought to be an important factor for optimizing the outcome of SCI. ^8^

Inflammation response in the spinal cord begins after macrophages activation and recruitment at the site of injury; activation of these macrophages could work in different pathways promoting, but also inhibiting the inflammation. Macrophages initiate inflammation by releasing pro-inflammatory cytokines (IL-1, IL-6, IL-8, IFN-γ and TNF-α) and other chemical mediators. After three or four days, macrophages that initiate and maintain the inflammation will then be deactivated by anti-inflammatory cytokines (IL-4, IL-10, IL-13 and TGF-β) that are also mainly produced by macrophages. The way that macrophages could regulate the inflammatory process is through the activation and counterbalance of pro- and anti-inflammatory cytokines. This balance determines the net effect of the inflammatory response in SCI. ^9^^,^^10^

ACTH _4-10_Pro ^8^-Gly ^9^-Pro ^10^ is an adrenocorticotropic hormone analogue that is known to have an anti-inflammatory effect. ^11^ It has also been used in many conditions such as stroke, traumatic brain injury and Alzheimer disease as a neuroprotective agent with no report of serious complications. ^12^

This experimental study was intended to show the effect of ACTH _4-10_ administration on anti-inflammatory cytokine levels in the spinal cord after acute SCI.

## Methods

In this study we used 12-week-old male Sprague Dawley rats weighing 250-300 g to reproduce homogenous sample for SCI models and eliminate confounding factor such as sex hormone. This animal study obtained ethical clearance by the Airlangga University animal care and use committee number 2.KE.094.07.2021 to ensure all effort was taken to reduce animal suffering. After randomization, 27 samples were divided into nine groups of three samples each. Laminectomy at the level of second thoracic vertebra was performed on all the samples. We took one group with the spinal cord left uninjured as a baseline control. The other samples became the treatment groups, in which SCI was performed by compressing the spine using an aneurysm clip in one minute with a clamping force of 20 g to reproduce mild SCI (four groups) and 35 g for severe SCI models (four groups). ^13^ The laminectomy site was then closed with sutures and the Sprague Dawley rats were kept alive.

After closing the suture, all treatment groups were divided into two subgroups. Placebo control groups were given 0.9 % NaCl intranasally, while the positive treatment groups were given ACTH _4-10_Pro ^8^-Gly ^9^-Pro ^10^ intranasally at a 300 mg/kg dose. Each group was divided again into two groups to be respectively terminated at 3 hours and 6 hours after compression. Myelum transection was performed at the level of injured myelum after the termination.

SCI myelum samples were fixed in 10% formalin and examined for IHC with specific monoclonal antibodies for each of IL-4, IL-10 and IL-13. Examination was done on the posterior horn of myelum to ensure homogenous sampling area in each group. Then anti-inflammatory cytokines were counted per 100 cells using associated anti-monoclonal antibodies and viewed with a light microscope at 1000× magnification. Cells with a brown colour in the cytoplasm showed positive expression of each anti-inflammatory cytokine.

The result of cytokine level measurements in each group are shown in a relative expression graph. Normality data analysis was performed using a Shapiro-Wilk test. Normally distributed data were analyzed with ANOVA while the data with non-normal distribution were analyzed using a Kruskal-Wallis test, followed by a non-parametric Mann-Whitney U test.

## Results

**Figure 1.**
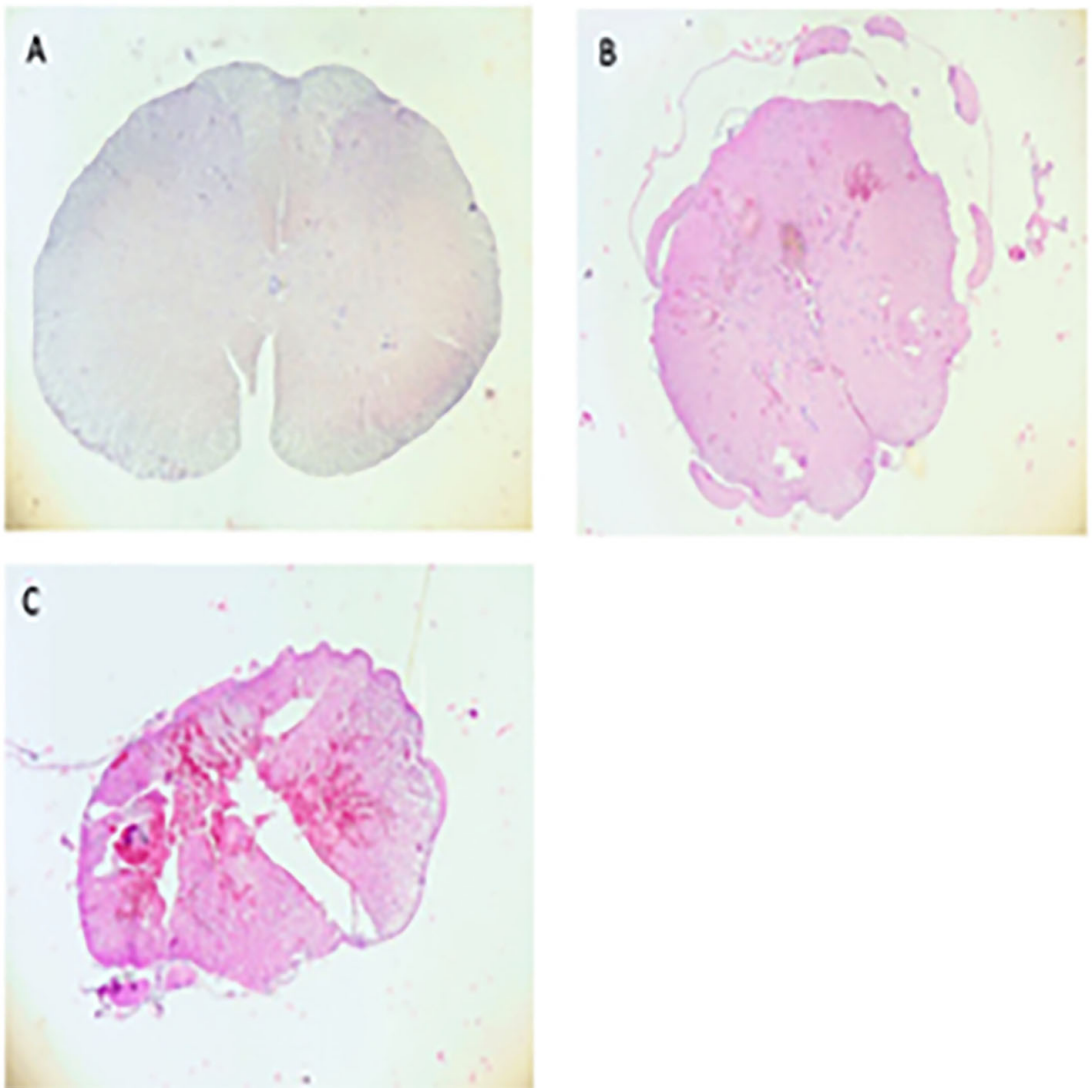
Myelum sample from control group (A), mild spinal cord injury (SCI, B), severe SCI (C).

We carried out an experimental study in 27 male Sprague Dawley rats weighing 250-300 g, we severed the spinal cords in the area of injury, then we fixed the specimen with formalin. The specimen was then cut into 4-6 μm-thick slices with rotatory microtome and mounted on glass slides before further staining as seen in Figure 1.

In Figure 1, the picture is without ACTH _4-10_Pro ^8^-Gly ^9^-Pro ^10^ administration after injury. This is expected so that the picture of myelum injury/damage looks different from mild to severe.

**Figure 2.**
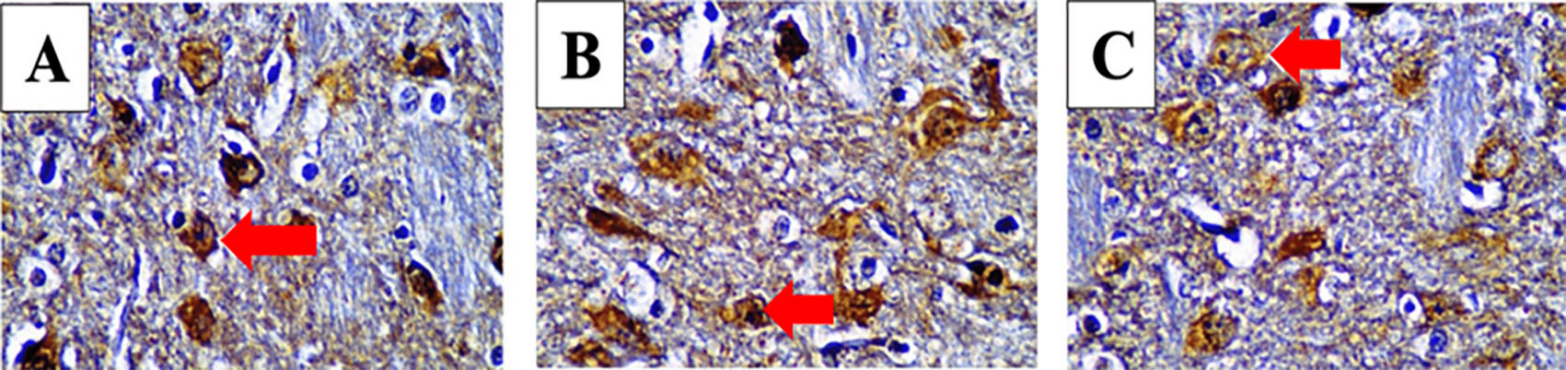
Cells were counted to assess anti-inflammatory cytokine levels in SCI models. ^37^ Immunoreacted neutrophil cytoplasm that stained brown after using monoclonal antibodies are shown. Red arrows mark positive stained neutrophils for IL-4 (A), IL-10 (B) and IL-13 (C) in 3 hours observed mild spinal cord injury (SCI) group.

After immunohistochemistry staining with each specific antibody we examined the posterior horn area and counted stained cell number per 100 cells with a light microscope at 1000× magnification. Positive cells appeared brown as seen in Figure 2.

**Figure 3.**
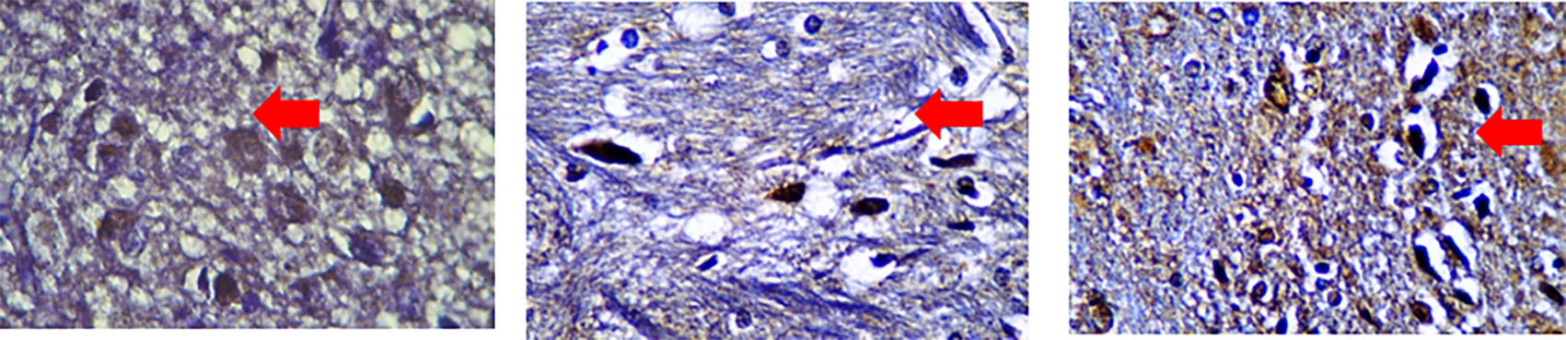
Cells were counted to assess anti-inflammatory cytokine levels in SCI models. ^37^ Immunoreacted neutrophil cytoplasm that stained brown after using monoclonal antibodies are shown. Red arrows mark positive stained neutrophils for IL-4 (A), IL-10 (B) and IL-13 (C) in 3 hours observed severe spinal cord injury (SCI) group.

The results Table 1 shows higher IL-4 levels in both mild and severe SCI models with ACTH _4-10_Pro ^8^-Gly ^9^-Pro ^10^ administration that were terminated after three and six hours, compared to the placebo group. Table 2 shows higher IL-10 levels in both mild and severe SCI models with ACTH _4-10_Pro ^8^-Gly ^9^-Pro ^10^ administration and who were terminated after three and six hours compared to the placebo group. Table 3 shows higher IL-13 levels in both mild and severe SCI models with ACTH _4-10_Pro ^8^-Gly ^9^-Pro ^10^ administration and who were terminates after three and six hours compared to the placebo group.

**Table 1.** ANOVA study of IL-4 levels. SCI: spinal cord injury.

Group	IL-4 expression		
	Mean	Standard deviation	p
Control	5.96	2.52	0.005 *
Mild SCI NaCl 0.9% 3 hours	6.29	3.40	
Mild SCI NaCl 0.9% 6 hours	5.61	2.45	
Mild SCI ACTH _4-10_ 3 hours	10.39	1.67	
Mild SCI ACTH _4-10_ 6 hours	8.05	1.62	
Control	5.75	2.20	0.001 *
Severe SCI NaCl 0.9% 3 hours	7.32	2.26	
Severe SCI NaCl 0.9% 6 hours	5.29	2.16	
Severe SCI ACTH _4-10_ 3 hours	9.36	1.98	
Severe SCI ACTH _4-10_ 6 hours	9.21	1.37	

* p < 0.05.

**Table 2.** ANOVA study of IL-10 levels.

Group	IL-10 expression		
	Mean	Standard deviation	p
Control	5.57	1.573	<0.001 *
Mild SCI NaCl 0.9% 3 hours	6.86	1.848	
Mild SCI NaCl 0.9% 6 hours	4.71	1.741	
Mild SCI ACTH _4-10_ 3 hours	14.64	2.893	
Mild SCI ACTH _4-10_ 6 hours	11.46	1.350	
Control	5.36	1.587	
Severe SCI NaCl 0.9% 3 hours	7.07	2.344	
Severe SCI NaCl 0.9% 6 hours	5.71	1.629	
Severe SCI ACTH _4-10_ 3 hours	13.25	3.086	
Severe SCI ACTH _4-10_ 6 hours	9.54	2.138	

* p < 0.05.

**Table 3.** ANOVA study of IL-13 level.

Group	IL-13 expression		
	Mean	Standard deviation	p
Control	3.86	1.682	<0.001 *
Mild SCI NaCl 0.9% 3 hours	7.86	2.445	
Mild SCI NaCl 0.9% 6 hours	6.25	2.046	
Mild SCI ACTH _4-10_ 3 hours	13.36	1.676	
Mild SCI ACTH _4-10_ 6 hours	11.57	3.313	
Control	4.50	1.893	
Severe SCI NaCl 0.9% 3 hours	5.79	1.410	
Severe SCI NaCl 0.9% 6 hours	5.68	1.718	
Severe SCI ACTH _4-10_ 3 hours	10.18	3.570	
Severe SCI ACTH _4-10_ 6 hours	9.00	1.893	

* p < 0.05.

Using Shapiro-Wilk test, we found our data had a normal distribution; further statistical analysis was carried out with a post hoc study using the Tukey method (Tables 4-9 in attachments).

Mean levels of IL4, IL-10 and IL-13 were higher in the mild SCI models group that was given ACTH _4-10_Pro ^8^-Gly ^9^-Pro ^10^ for both three- and six-hour termination groups, compared with placebo. IL-14 levels in models given ACTH _4-10_Pro ^8^-Gly ^9^-Pro ^10^ at three and six hours were 10.39 and 8.05 respectively, and 6.29 and 5.61 respectively in the placebo group. IL-10 levels in ACTH _4-10_Pro ^8^-Gly ^9^-Pro ^10^ at three and six hours were 14.64 and 11.46 respectively, while in the placebo group they were 6.86 and 4.71 respectively. IL-13 levels in ACTH _4-10_Pro ^8^-Gly ^9^-Pro ^10^ at three and six hours were 13.36 and 11.57 respectively, and 7.86 and 6.25 respectively in the placebo group. Post hoc analysis with Tukey test showed significant differences in IL-4, IL-10 and IL-13 at three hours, and in IL-10 and IL-13 at six hours.

**Figure 4.**
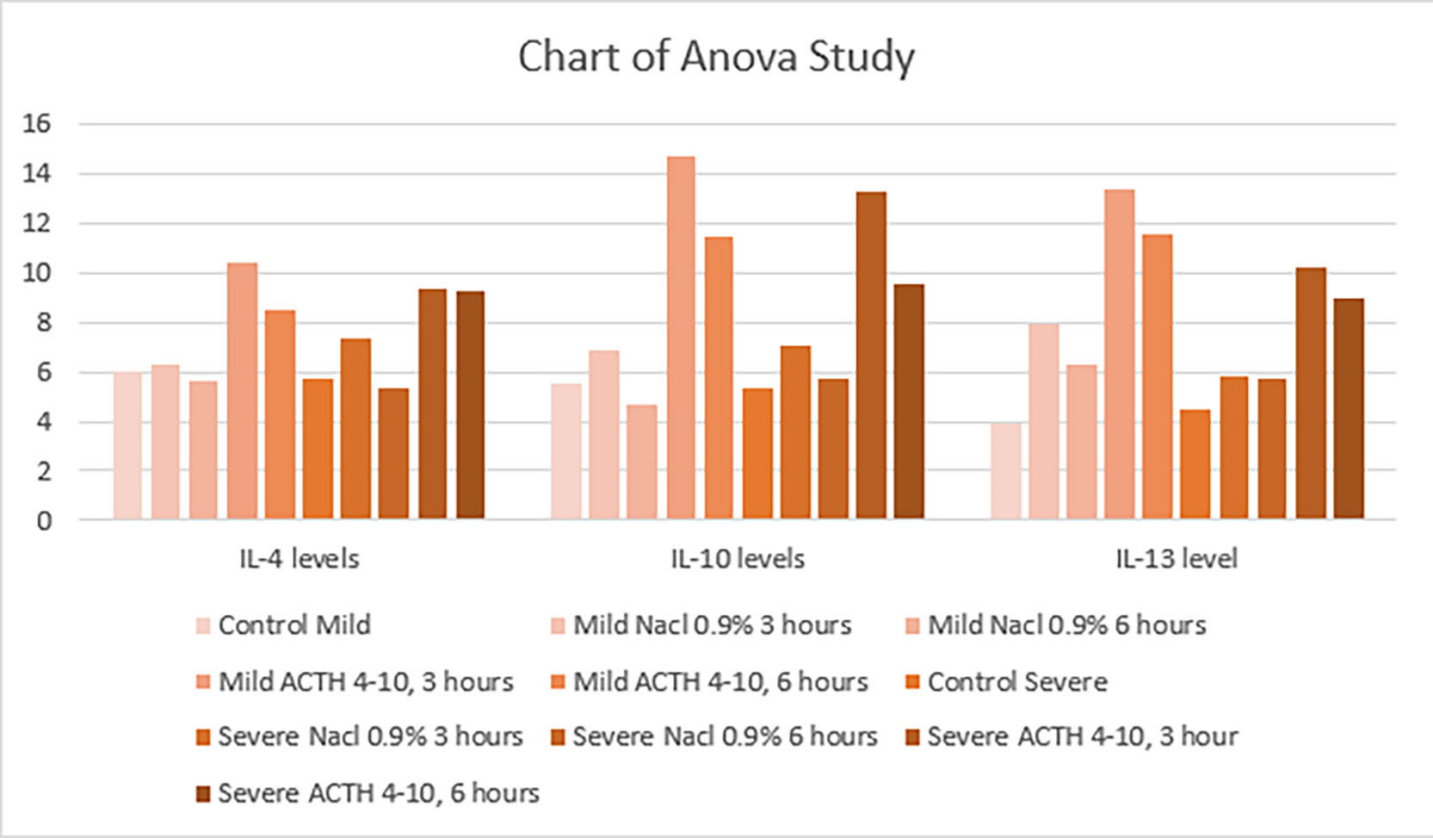
Chart of Annova Study.

Figure 4 show results from severe SCI models that were given ACTH _4-10_Pro ^8^-Gly ^9^-Pro ^10^ also showed higher mean IL-4, IL-10 and IL-13 levels compared with placebos in the three-hour and six-hour groups. IL-4 levels in ACTH _4-10_Pro ^8^-Gly ^9^-Pro ^10^ at three and six hours were 9.36 and 9.21 respectively, and 7.82 and 5.29 respectively in the placebo. IL-10 levels in ACTH _4-10_Pro ^8^-Gly ^9^-Pro ^10^ at three and six hourswas 13.25 and 9.54 respectively, while in placebos they were 7.07 and 5.71, respectively. IL-13 levels in models administered ACTH _4-10_Pro ^8^-Gly ^9^-Pro ^10^ at three and six hours were 10.18 and 9.00 respectively, while in placebo they were 5.79 and 5.68 respectively. *Post hoc* analysis with Tukey test showed significant differences in IL-10 and IL-13 levels at three hours, and in IL-4 and IL-10 at six hours. Overall, level of anti-inflammatory cytokines were higher in each treatment group compared with placebo; the levels were significantly higher in models with mild SCI and terminated three hours after injury.

## Discussion

Inflammation following the primary spinal cord injury is known to cause further spinal cord damage, resulting in apoptosis of oligodendrocyte, demyelination and degradation of axons, and neuronal cell death. Many attempts were made to reduce this deleterious effect caused by inflammation after initial injury occurrs. ^14^

Neuroinflammation following the primary injury is predominantly regulated by the balance of macrophage activation. ^15^ In order to regulate this inflammation cascade, macrophages play a key role by releasing pro- or anti-inflammatory cytokine. Studies in SCI models have shown that some pro-inflammatory cytokines like TNF-α, IL-1, and IL-6 are found upregulated within an hour after injury, leading to further inflammatory response causing neuronal apoptosis. ^16^ In contrast, anti-inflammatory cytokines that are capable of altering macrophage activity, such as IL-4, IL-13 ^16^^–^^19^ and IL-10 which can directly inhibiting other pro-inflammatory cytokines, are generally present at low levels after SCI. ^20^^,^^21^

We realize sex hormone like estrogen and progesterone have some immunosuppressive profile in SCI ^22^; to minimize this potentially confounding factor that might bias the outcome we only used male Sprague Dawley rats in this study. Our study found significantly higher IL-4, IL-10 and IL-13 levels in the mild SCI group that was terminated after three hours, and higher IL-10, IL-13 levels in the six-hour termination group that was given ACTH _4-10_. In the severe SCI group that were given ACTH _4-10_, we found higher IL-10, IL-13 levels in the group that was terminated after three hours, and higher IL-4 and IL-10 levels after six hours.

Compared with mild SCI, in the severe SCI group inflammation was more pronounced and abundant in pro-inflammatory cytokines. These pro-inflammatory cytokines in turn could suppress the release of anti-inflammatory cytokines. In addition, inflammation that occurs in SCI rat models is known to begin one hour after injury and reach its peak at six hours after injury. ^23^ This is in accordance with our study, that the anti-inflammatory cytokine group from the mild or severe injury group showed an increase in the ratio of inflammatory cytokines at 3 to 6 hours. However, in the type of inflammatory cytokine IL-10, the peak cytokine ratio was at 3 hours, and decreased at 6 hours. The decrease in the inflammatory cytokine IL-10 at 6 hours is possibly due to a decrease in the influence of ACTH _4-10_Pro ^8^-Gly ^9^-Pro ^10^ at 6 hours. After the strongest influence to increase cytokines at 3 hours.

ACTH _4-10_Pro ^8^-Gly ^9^-Pro ^10^ is an analogue of adrenocorticotropic hormone and is created from a fragment of ACTH(4–7) combined with C-terminal tripeptide Pro-Gly-Pro, which acts as a neuroprotective compound. ^24^ Similar to its analogue, ACTH _4-10_Pro ^8^-Gly ^9^-Pro ^10^ is said to bind at the melanocortin receptor promoting anti-inflammatory and neuroprotective effects. Activated melanocortin receptors could supress pro-inflammatory cytokine release and promote the anti-inflammatory cytokine release. Together, this condition could supress inflammation. ^26^ In another study, when compared with the administration of steroids, that Methylprednisolone could attenuate astrocyte cell death, decrease microglia activation, suppress A1 astrocytes activation, and promote functional recovery after acute SCI in mouse models. ^25^

Methylprednisolone sodium succinate (MP) became the most discussed treatment in the last three to four decades for reducing the deleterious effect caused by inflammation after initial injury occurs. The first study that was conducted in the early 1980s showed a promising potential neuroprotective effect of MP in reducing inflammation after acute SCI in a preclinical setting. This study has led many clinicians to do more extensive research (NASCIS I and II). ^27^^,^^28^ This large-scale randomized control trial showed that there was a statistical improvement in neurological outcomes in the treatment group compared with the placebo if MP was administered within less than eight hours after initial injury. ^28^^,^^29^ However, with an average increase of just five points in the American Spinal Injury Association (ASIA) impairment score compared with the placebo, this was said to not be clinically significant in the overall quality of life of the patient. Moreover, along with the limited overall improvement, a high dose of MP came with several complications, such as increased rate of infection along with gastrointestinal haemorrhages. ^30^^,^^31^ Considering the limited benefit and potential morbidity or even life-threatening complications, in 2013 the American Association of Neurological Surgeon (AANS) ruled out MP administration from the current recommendations for managing patients with SCI. ^32^^,^^33^

Our finding showing increased levels of anti-inflammatory cytokines in the treatment group complements previous studies that revealed ACTH _4-10_Pro ^8^-Gly ^9^-Pro ^10^ could decrease pro-inflammatory cytokines in SCI rats model. ^34^^,^^35^ Those studies might provide a fundamental basis showing ACTH _4-10_Pro ^8^-Gly ^9^-Pro ^10^ could reduce inflammation and potentially prevent secondary injury in SCI. To date, ACTH _4-10_Pro ^8^-Gly ^9^-Pro ^10^ has been used as a neuroprotective agent for stroke and Alzheimer patients with no report of serious complications. It has also been given through the intranasal route and has a rapid onset within minute, allowing this drug to be given early on after the initial injury. Finally, Increased anti-inflammatory mediators can be triggered by ACTH _4-10_Pro ^8^-Gly ^9^-Pro ^10^, which is expected to suppress inflammatory events that can trigger further secondary injury.

## Conclusions

Administration of ACTH _4-10_Pro ^8^-Gly ^9^-Pro ^10^ can increase anti-inflammatory cytokine levels in Sprague Dawley rat models with mild and severe SCI. The optimal effect of ACTH _4-10_Pro ^8^-Gly ^9^-Pro ^10^ can increase anti-inflammatory cytokine occurs in the third hour after SCI injury. Further studies are needed to determine the optimal dose and functional outcome in rats with SCI.

## Figures and Tables

**Table 4.** Post hoc study of IL-4 level in mild SCI.

Group	Mean	Standard deviation	Group	Mean	Standard deviation	p
Control	5.96	2.52	NaCl 0.9% 3 hours	6.29	3.40	0.999
			NaCl 0.9% 6 hours	5.61	2.45	0.999
			ACTH _4-10_ 3 hours	10.39	1.67	0.015 *
			ACTH _4-10_ 6 hours	8.05	1.62	0.510
NaCl 0.9% 3 hour	6.29	3.40	NaCl 0.9% 6 hours	5.61	2.45	0.984
			ACTH _4-10_ 3 hours	10.39	1.67	0.027 *
			ACTH _4-10_ 6 hours	8.05	1.62	0.663
NaCl 0.9% 6 hour	5.61	2.45	ACTH _4-10_ 3 hours	10.39	1.67	0.007 *
			ACTH _4-10_ 6 hours	8.05	1.62	0.353
ACTH _4-10_ 3 hour	10.39	1.67	ACTH _4-10_ 6 hours	8.04	1.62	0.382

* p < 0.05.

**Table 5.** *Post hoc* study of IL-10 level in mild SCI.

Group	Mean	Standard deviation	Group	Mean	Standard deviation	p
Control	5.57	1.573	NaCl 0.9% 3 hours	6.86	1.848	0.734
			NaCl 0.9% 6 hours	4.71	1.741	0.922
			ACTH _4-10_ 3 hours	14.64	2.893	<0.001 *
			ACTH _4-10_ 6 hours	11.46	1.350	<0.001 *
NaCl 0.9% 3 hour	6.86	1.848	NaCl 0.9% 6 hours	4.71	1.741	0.268
			ACTH _4-10_ 3 hours	14.64	2.893	<0.001 *
			ACTH _4-10_ 6 hours	11.46	1.350	<0.001 *
NaCl 0.9% 6 hour	4.71	1.741	ACTH _4-10_ 3 hours	14.64	2.893	<0.001 *
			ACTH _4-10_ 6 hours	11.46	1.350	<0.001 *
ACTH _4-10_ 3 hour	14.64	2.893	ACTH _4-10_ 6 hours	11.46	1.350	0.036 *

* p < 0.05.

**Table 6.** *Post hoc* study of IL-13 level in mild SCI.

Group	Mean	Standard deviation	Group	Mean	Standard deviation	p
Control	3.86	1.682	NaCl 0.9% 3 hours	7.86	2.445	0.023 *
			NaCl 0.9% 6 hours	6.25	2.046	0.322
			ACTH _4-10_ 3 hours	13.36	1.676	<0.001 *
			ACTH _4-10_ 6 hours	11.57	3.313	<0.001 *
NaCl 0.9% 3 hour	7.86	2.445	NaCl 0.9% 6 hours	6.25	2.046	0.694
			ACTH _4-10_ 3 hours	13.36	1.676	0.001 *
			ACTH _4-10_ 6 hours	11.57	3.313	0.040 *
NaCl 0.9% 6 hour	6.25	2.046	ACTH _4-10_ 3 hours	13.36	1.676	<0.001 *
			ACTH _4-10_ 6 hours	11.57	3.313	0.001 *
ACTH _4-10_ 3 hour	13.36	1.676	ACTH _4-10_ 6 hours	11.57	3.313	0.605

* p < 0.05.

**Table 7.** *Post hoc* study of IL-4 level in severe SCI.

Group	Mean	Standard deviation	Group	Mean	Standard deviation	p
Control	5.75	2.20	NaCl 0.9% 3 hours	7.32	2.26	0.600
			NaCl 0.9% 6 hours	5.29	2.16	0.993
			ACTH _4-10_ 3 hours	9.36	1.98	0.018 *
			ACTH _4-10_ 6 hours	9.21	1.37	0.025 *
NaCl 0.9% 3 hour	7.32	2.26	NaCl 0.9% 6 hours	5.29	2.16	0.349
			ACTH _4-10_ 3 hours	9.36	1.98	0.349
			ACTH _4-10_ 6 hours	9.21	1.37	0.421
NaCl 0.9% 6 hour	5.29	2.16	ACTH _4-10_ 3 hours	9.36	1.98	0.006 *
			ACTH _4-10_ 6 hours	9.21	1.37	0.009 *
ACTH _4-10_ 3 hour	9.36	1.98	ACTH _4-10_ 6 hours	9.21	1.37	1.000

* p < 0.05.

**Table 8.** *Post hoc* study of IL-10 level in severe SCI.

Group	Mean	Standard deviation	Group	Mean	Standard deviation	p
Control	5.36	1.587	NaCl 0.9% 3 hours	7.07	2.344	0.607
			NaCl 0.9% 6 hours	5.71	1.629	0.998
			ACTH _4-10_ 3 hours	13.25	3.086	<0.001 *
			ACTH _4-10_ 6 hours	9.54	2.138	0.012
NaCl 0.9% 3 hour	7.07	2.344	NaCl 0.9% 6 hours	5.71	1.629	0.784
			ACTH _4-10_ 3 hours	13.25	3.086	<0.001 *
			ACTH _4-10_ 6 hours	9.54	2.138	0.258
NaCl 0.9% 6 hour	5.71	1.629	ACTH _4-10_ 3 hours	13.25	3.086	<0.001 *
			ACTH _4-10_ 6 hours	9.54	2.138	0.024 *
ACTH _4-10_ 3 hour	13.25	3.086	ACTH _4-10_ 6 hours	9.54	2.138	0.030 *

* p < 0.05.

**Table 9.** *Post hoc* study of IL-13 level in severe SCI.

Group	Mean	Standard deviation	Group	Mean	Standard deviation	p
Control	4.50	1.893	NaCl 0.9% 3 hours	5.79	1.410	0.816
			NaCl 0.9% 6 hours	5.68	1.718	0.858
			ACTH _4-10_ 3 hours	10.18	3.570	<0.001
			ACTH _4-10_ 6 hours	9.00	1.893	0.006 *
NaCl 0.9% 3 hour	5.79	1.410	NaCl 0.9% 6 hours	5.68	1.718	1.000
			ACTH _4-10_ 3 hours	10.18	3.570	0.007 *
			ACTH _4-10_ 6 hours	9.00	1.893	0.078
NaCl 0.9% 6 hour	5.68	1.718	ACTH _4-10_ 3 hours	10.18	3.570	0.006 *
			ACTH _4-10_ 6 hours	9.00	1.893	0.064
ACTH _4-10_ 3 hour	10.18	3.570	ACTH _4-10_ 6 hours	9.00	1.893	0.858

* p < 0.05.

## Data Availability

DRYAD: Effects of ACTH4-10Pro8-Gly9-Pro10 on anti-inflammatory cytokine (IL-4, IL-10, IL-13) expression in acute spinal cord injury model (Sprague Dawley rats), https://doi.org/10.5281/zenodo.7446188. ^36^ Zenodo: Effects of ACTH4-10Pro8-Gly9-Pro10 on anti-inflammatory cytokine (IL-4, IL-10, IL-13) expression in acute spinal cord injury model (Sprague Dawley rats), https://doi.org/10.5281/zenodo.7568360. ^37^ Zenodo: ARRIVE checklist for “Effect of ACTH4-10Pro8-Gly9-Pro10 on anti-inflammatory cytokine (IL-4, IL-10, IL-13) expression in acute spinal cord injury models (male Sprague Dawley rats)”, https://doi.org/10.5281/zenodo.7568360. ^37^ Data are available under the terms of the Creative Commons Attribution 4.0 International Public License (CC-BY 4.0).
