# Supplementary material for: Effect of ACTH4-10Pro8-Gly9-Pro10 on anti-inflammatory cytokine (IL-4, IL-10, IL-13) expression in acute spinal cord injury models (male Sprague Dawley rats)
Source: F1000Res. 2025 Sep 22;12:194. Originally published 2023 Feb 20. [Version 2] doi: 10.12688/f1000research.127413.2 (PMC12579320; doi:10.12688/f1000research.127413.2)
Supplement: Peer review history

# Reviewer report: Reviewer response for version 2

**DOI:** 10.5256/f1000research.177886.r416146

**Published:** 2025-10-31

**Authors:** Mawaddah Ar Rochmah

**Affiliations:** 1 Department of Neurology Faculty of Medicine, Public Health, and Nursing, Universitas Gadjah Mada, Yogyakarta, Indonesia

The authors have made the necessary revisions in the current manuscript.

Is the work clearly and accurately presented and does it cite the current literature?

Yes

If applicable, is the statistical analysis and its interpretation appropriate?

Yes

Are all the source data underlying the results available to ensure full reproducibility?

Partly

Is the study design appropriate and is the work technically sound?

Yes

Are the conclusions drawn adequately supported by the results?

Yes

Are sufficient details of methods and analysis provided to allow replication by others?

Partly

Reviewer Expertise:

Neurology, Genetic

I confirm that I have read this submission and believe that I have an appropriate level of expertise to confirm that it is of an acceptable scientific standard.

# Reviewer report: Reviewer response for version 2

**DOI:** 10.5256/f1000research.177886.r423811

**Published:** 2025-10-29

**Authors:** Xue Yao

**Affiliations:** 1 Tianjin Key Laboratory of Spine and Spinal Cord, Tianjin, China

This article focuses on the core link after spinal cord injury - neuroinflammation. It selects ACTH analogues with no serious complications reported and adopts the non-invasive, fast-acting intranasal administration method suitable for early intervention in acute injury, which has high clinical transformation feasibility. However, some issues may be addressed to improve the current manuscript.

1. The preface section describes that the pro-inflammatory macrophages are inactivated by anti-inflammatory cytokines IL-4 and IL-10 three to four days after spinal cord injury. Why the experimental design does not choose to continue the repeated detection of IL-4, IL-10 and IL-13 until three to four days, but only selects 3 hours and 6 hours? It is suggested to provide supplementary explanations.
2. Figure 1 is blurry and of poor quality. It is recommended to replace it with a clearer image.
3. It is suggested that the tables in Table 1-3 be transformed into bar charts and placed on Figures 2 and 3, which will enhance the readability of the article for readers.
4. In the sixth paragraph of the results section, the author mistakenly wrote IL4 as IL14. There was an error in the spelling of a proper noun. It is recommended that you read the entire text carefully and conduct a thorough review of the entire article.
5. In the writing of research papers, * indicates P < 0.05, ** indicates P < 0.01, and *** indicates P < 0.001. It is recommended to provide supplementary statistical results for the tables in the article.
6. The sample size of the experiment was relatively small, with only 3 rats in each group. Although the differences were confirmed to be significant through statistical tests, the stability and generalizability of the results require verification with a larger sample size.
7. Without excluding the indirect influence of gender-related confounding factors, although male rats were used to avoid the interference of neuroprotective effects of estrogen and progesterone, the influence of gender differences on the ACTH _4-10_ effect was not explored. The results are only applicable to the male model, and additional verification with female animals is required for clinical application.
8. The mechanism of action has not been thoroughly investigated. It is only clear that ACTH _4-10 _can upregulate anti-inflammatory factors, but the molecular mechanism has not been revealed: such as whether it functions by binding to the melanocyte-stimulating hormone receptor (MC receptor), whether it regulates downstream signaling pathways (such as NF-κB), whether the increase in anti-inflammatory factors is directly induced by the drug rather than a compensatory response after injury, etc. The mechanism analysis remains at the level of phenomenon description.

Is the work clearly and accurately presented and does it cite the current literature?

No

If applicable, is the statistical analysis and its interpretation appropriate?

No

Are all the source data underlying the results available to ensure full reproducibility?

No

Is the study design appropriate and is the work technically sound?

No

Are the conclusions drawn adequately supported by the results?

No

Are sufficient details of methods and analysis provided to allow replication by others?

No

Reviewer Expertise:

spinal cord injury

I confirm that I have read this submission and believe that I have an appropriate level of expertise to state that I do not consider it to be of an acceptable scientific standard, for reasons outlined above.

# Reviewer report: Reviewer response for version 2

**DOI:** 10.5256/f1000research.177886.r423815

**Published:** 2025-10-25

**Authors:** Cafer Ikbal Gulsever

**Affiliations:** 1 İstanbul University, İstanbul, Turkey

- **Scientific merit:**
  The study addresses a relevant and novel topic — the anti-inflammatory role of ACTH4-10Pro8-Gly9-Pro10 (Semax) in acute spinal cord injury. The experimental design using Sprague-Dawley rats is appropriate, and the methodology (clip-compression model, immunohistochemistry, ANOVA analysis) is sound.
- **Data and presentation:**
  Figures and statistical results are clear and well-organized after revision. The inclusion of IL-4, IL-10, and IL-13 as markers strengthens the biological interpretation.
- **Revisions adequately addressed:**
  The authors have responded thoroughly to the previous reviewer’s comments—clarifying dosage, source of reagents, timing of administration, and improving figures and statistical visualization.
- **Writing and clarity:**
  The manuscript is generally well written. Minor linguistic polishing may further improve readability, but does not affect scientific quality.
- **Recommendation:**
  The article is scientifically solid, revisions are satisfactory, and data are consistent with conclusions.
- **Recommendation: Accept (minor editorial corrections only).**

Is the work clearly and accurately presented and does it cite the current literature?

Yes

If applicable, is the statistical analysis and its interpretation appropriate?

Yes

Are all the source data underlying the results available to ensure full reproducibility?

Yes

Is the study design appropriate and is the work technically sound?

Yes

Are the conclusions drawn adequately supported by the results?

Yes

Are sufficient details of methods and analysis provided to allow replication by others?

Yes

Reviewer Expertise:

Spinal cord injury, emergency neurosurgery

I confirm that I have read this submission and believe that I have an appropriate level of expertise to confirm that it is of an acceptable scientific standard.

# Reviewer report: Reviewer response for version 1

**DOI:** 10.5256/f1000research.139917.r164062

**Published:** 2023-03-13

**Authors:** Mawaddah Ar Rochmah

**Affiliations:** 1 Department of Neurology Faculty of Medicine, Public Health, and Nursing, Universitas Gadjah Mada, Yogyakarta, Indonesia

This study explored the use of ACTH4-10Pro8-Gly9-Pro10 in the rat model of spinal cord injury (SCI). The study focused on the role of the above-mentioned agent in ameliorating neuroinflammation within the acute phase of the SCI by measuring the inflammatory cytokines after the treatments. The study is novel and well-written. However, there are some issues that might be addressed to improve the current manuscript.

 The introduction is quite informative to provide the background of the study. However, in the introduction section, the authors are suggested to elaborate on the introduction of the ACTH4-10Pro8-Gly9-Pro10 in the treatment of SCI.

 There is information that the authors need to provide in the materials & methods section. In the methods section, it would be clearer if the authors completed the following pieces of information:

- Why was the ACTH4-10Pro8-Gly9-Pro10 intranasally at a 300 mg/kg dose chosen? Any previous study that suggested the dose? If any, please provide the reference and citation.
- How long was the period between treatment initiation after finishing the laminectomy procedure?
- Please provide the companies which produce the materials (reagents, software, etc) for the purpose of reproducibility.
- Please kindly provide the information if the measurement was performed at least in duple for each sample.

These are some suggestions for the results section (including figures and tables):

- The authors are suggested to provide more description of Figure 1, either in Figure Legend of Figure 1 or as a narration in the text. Figure 1 is the evidence to differentiate the spinal cord in healthy, mild SCI, and severe SCI. Please provide the information on whether the histological figure of the spinal cord SCI groups was with or without the administration of  ACTH4-10Pro8-Gly9-Pro10 intranasally. The authors need to provide the readers with what to expect in the microscopic changes of the spinal cord after mild and severe SCI.
- In Figure 2, the white arrows are not quite readily visible, the authors are suggested to pick a more contrasting color for the arrow. Why did the authors show only the mild SCI groups here? The authors are suggested to present at least one representative figure for each IHC staining for every group.
- Tables 1-3 are best presented in bar diagrams between groups in a figure. This will give the readers a better description and impression of the results of the study. In addition, the authors can explain more detailed data in the text. This will avoid the repetition of data explanation in figures/tables in the text.
- Tables 4-9, in my opinion, can be submitted as supplementary data. In the results section, the authors are suggested to explain the core findings of the posthoc analysis in the text.
- The authors are suggested to make sure that figures and tables are self-explanatory. Any repetition of the data from the figure/tables in the text should be avoided.

In the discussion section, the authors have explained and compared the results of the study to other previous studies.

 Please kindly focus on the difference between the follow up period of 3 hrs and 6 hrs in the study, particularly in the level of the anti-inflammatory cytokines and the SCI severity.

 In my opinion, I found the fifth paragraph of this section a little odd since the authors did not mention that the study using methylprednisolone was performed in humans. If the authors would like to compare the efficacy of the currently used treatment of SCI, in this case, is methylprednisolone, to the agent used here in the study, I would prefer to use the studies with the same subjects (in this case, the authors should also add any study regarding the use of methylprednisolone treatment in rodents. The study in humans should come as an additional information), the same studied objects/variables (any agents that may increase or decrease anti-inflammatory cytokine in SCI, neurological deficit severity, etc), or other agents that have similar mechanisms of action (in this case, any other agent that binds to melanocortin receptor).

 In the last sentence of the discussion section, the authors mention “Finally, we see potential benefits from ACTH4-10Pro8- Gly9-Pro10 to reduce inflammation and its deleterious effect on secondary injury after SCI”. Please kindly provide the “deleterious effect of the SCI” that was studied here, since the anti-inflammatory cytokines studied here, in my understanding, are not the deleterious effects of the SCI.

 The authors are suggested to make the conclusion brief indicating that anti-inflammatory cytokines might be increased after the administration of ACTH4-10Pro8-Gly9-Pro10 intranasally in the acute SCI model.

Is the work clearly and accurately presented and does it cite the current literature?

Yes

If applicable, is the statistical analysis and its interpretation appropriate?

Yes

Are all the source data underlying the results available to ensure full reproducibility?

Partly

Is the study design appropriate and is the work technically sound?

Yes

Are the conclusions drawn adequately supported by the results?

Yes

Are sufficient details of methods and analysis provided to allow replication by others?

Partly

Reviewer Expertise:

Neurology, Genetic

I confirm that I have read this submission and believe that I have an appropriate level of expertise to confirm that it is of an acceptable scientific standard, however I have significant reservations, as outlined above.

# Response

**Published:** 2024-10-04

**Authors:** Asadullah Asadullah

**Affiliations:** Airlangga University, Indonesia

- Why was the ACTH4-10Pro8-Gly9-Pro10 intranasally at a 300 mg/kg dose chosen? Any previous study that suggested the dose? If any, please provide the reference and citation.

>This is previous study https://www.scirp.org/journal/paperinformation.aspx?paperid=40560 . Dose is 300 mcg / kg. not mg. thank you. 

- How long was the period between treatment initiation after finishing the laminectomy procedure?

> administration of drugs after 1 hour of laminectomy and spinal cord clip with aneurysm clip to reproduce spinal cord injury.

- Please provide the companies which produce the materials (reagents, software, etc) for the purpose of reproducibility.

> The medicine is from PT Axomedica with the trade name Semax

# Response

**Published:** 2025-07-23

**Authors:** Asadullah Asadullah

**Affiliations:** Airlangga University, Indonesia

Note Of revision: 

 Revision on Method:

 •    Why was the ACTH4-10Pro8-Gly9-Pro10 intranasally at a 300 mg/kg dose chosen? Any previous study that suggested the dose? If any, please provide the reference and citation.

 Rev: 

 After closing the suture, all treatment groups were divided into two subgroups. Placebo control groups were given 0.9 % NaCl intranasally, while the positive treatment groups were given ACTH4-10Pro 8-Gly 9-Pro 10 or semax (The medicine is from PT Axomedica) intranasally at a 300 mcg/kg dose.38  

 38. Kolomin, T., Shadrina, M., Slominsky, P., Limborska, S., & Myasoedov, N. (2013). A New Generation of Drugs: Synthetic Peptides Based on Natural Regulatory Peptides. Neuroscience and Medicine, 04(04), 223–252. https://doi.org/10.4236/nm.2013.44035

 •    How long was the period between treatment initiation after finishing the laminectomy procedure?

**Rev:**

 Administration of semax after 1 hour of laminectomy and spinal cord clip with aneurysm clip to reproduce spinal cord injury.

 •    Please provide the companies which produce the materials (reagents, software, etc) for the purpose of reproducibility.

**Rev:**

 The medicine is from PT Axomedica with the trade name Semax

 •    Please kindly provide the information if the measurement was performed at least in duple for each sample.

**Rev:**

 Each group was divided again into two groups to be respectively terminated at 3 hours and 6 hours after compression. Myelum transection was performed at the level of injured myelum after the termination.

 These are some suggestions for the results section (including figures and tables):

 •    The authors are suggested to provide more description of Figure 1, either in Figure Legend of Figure 1 or as a narration in the text. Figure 1 is the evidence to differentiate the spinal cord in healthy, mild SCI, and severe SCI. Please provide the information on whether the histological figure of the spinal cord SCI groups was with or without the administration of  ACTH4-10Pro8-Gly9-Pro10 intranasally. The authors need to provide the readers with what to expect in the microscopic changes of the spinal cord after mild and severe SCI.

 link of figure 1 : https://drive.google.com/file/d/1dIOgMAQP9lsSUdSw8y3vYRLLfnjYeIGs/view?usp=sharing

**Rev:**

 In Figure 1, the picture is without ACTH4-10Pro8-Gly9-Pro10 administration after injury. This is expected so that the picture of myelum injury/damage looks different from mild to severe.

 link of figure 1 : https://drive.google.com/file/d/1dIOgMAQP9lsSUdSw8y3vYRLLfnjYeIGs/view?usp=sharing

**Rev:**

 Figure 2. Cells were counted to assess anti-inflammatory cytokine levels in SCI models. 37  Immunoreacted neutrophil cytoplasm that stained brown after using monoclonal antibodies are shown. White arrows mark positive stained neutrophils for IL-4 (A), IL-10 (B) and IL-13 (C) in  3 hours observed mild spinal cord injury (SCI) group.

 Link of figure 2: https://drive.google.com/file/d/1zxPR-is_fgwmhZ_BKLKsHjCAd8jgLaAs/view?usp=sharing

 Figure 3. Cells were counted to assess anti-inflammatory cytokine levels in SCI models. 37  Immunoreacted neutrophil cytoplasm that stained brown after using monoclonal antibodies are shown. White arrows mark positive stained neutrophils for IL-4 (A), IL-10 (B) and IL-13 (C) in  3 hours observed severe spinal cord injury (SCI) group.

 LInk of figure 3: https://drive.google.com/file/d/10G-8kJvhFzgFQYzp7uQOwu4CnPNmLv8B/view?usp=sharing

 •    Tables 1-3 are best presented in bar diagrams between groups in a figure. This will give the readers a better description and impression of the results of the study. In addition, the authors can explain more detailed data in the text. This will avoid the repetition of data explanation in figures/tables in the text.

 Rev:

 Figure 4. Chart Of Annova Study

 link of  figure 4: https://drive.google.com/file/d/1DcLsen6EXCZBj-na9Wpg-qkHWc9gcv1r/view?usp=sharing

 •    Tables 4-9, in my opinion, can be submitted as supplementary data. In the results section, the authors are suggested to explain the core findings of the posthoc analysis in the text.

**Rev**: Done. Tables had deleted, and change as as supplementary data.

 •    The authors are suggested to make sure that figures and tables are self-explanatory. Any repetition of the data from the figure/tables in the text should be avoided.

**Rev**: Done

 •    In the discussion section, the authors have explained and compared the results of the study to other previous studies.

 •    Please kindly focus on the difference between the follow up period of 3 hrs and 6 hrs in the study, particularly in the level of the anti-inflammatory cytokines and the SCI severity.

 •    In my opinion, I found the fifth paragraph of this section a little odd since the authors did not mention that the study using methylprednisolone was performed in humans. If the authors would like to compare the efficacy of the currently used treatment of SCI, in this case, is methylprednisolone, to the agent used here in the study, I would prefer to use the studies with the same subjects (in this case, the authors should also add any study regarding the use of methylprednisolone treatment in rodents. The study in humans should come as an additional information), the same studied objects/variables (any agents that may increase or decrease anti-inflammatory cytokine in SCI, neurological deficit severity, etc), or other agents that have similar mechanisms of action (in this case, any other agent that binds to melanocortin receptor).

**Rev:**

 This is in accordance with our study, that the anti-inflammatory cytokine group from the mild or severe injury group showed an increase in the ratio of inflammatory cytokines at 3 to 6 hours. However, in the type of inflammatory cytokine IL-10, the peak cytokine ratio was at 3 hours, and decreased at 6 hours. The decrease in the inflammatory cytokine IL-10 at 6 hours is possibly due to a decrease in the influence of ACTH 4-10Pro 8-Gly 9-Pro 10 at 6 hours. After the strongest influence to increase cytokines at 3 hours.

 In another study, when compared with the administration of steroids, that Methylprednisolone could attenuate astrocyte cell death, decrease microglia activation, suppress A1 astrocytes activation, and promote functional recovery after acute SCI in mouse models.

 Finally, Increased anti-inflammatory mediators can be triggered by ACTH4-10Pro 8- Gly 9-Pro 10, which is expected to suppress inflammatory events that can trigger further secondary injury.

 •    In the last sentence of the discussion section, the authors mention “Finally, we see potential benefits from ACTH4-10Pro8- Gly9-Pro10 to reduce inflammation and its deleterious effect on secondary injury after SCI”. Please kindly provide the “deleterious effect of the SCI” that was studied here, since the anti-inflammatory cytokines studied here, in my understanding, are not the deleterious effects of the SCI.

**Rev:**

 Administration of ACTH 4-10Pro 8-Gly 9-Pro 10 can increase anti-inflammatory cytokine levels in Sprague Dawley rat models with mild and severe SCI. The optimal effect of ACTH 4-10Pro 8-Gly 9-Pro 10 can increase anti-inflammatory cytokine occurs in the third hour after SCI injury.  Further studies are needed to determine the optimal dose and functional outcome in rats with SCI.
